# (μ-Piperazine-1,4-dicarbodithio­ato-κ^4^ *S* ^1^,*S* ^1′^:*S* ^4^,*S* ^4′^)bis­[bis­(triphenyl­phos­phane-κ*P*)gold(I)] chloro­form disolvate

**DOI:** 10.1107/S1600536811044229

**Published:** 2011-10-29

**Authors:** Ilia A. Guzei, Lara C. Spencer, Stacy Lillywhite, James Darkwa

**Affiliations:** a Department of Chemistry, University of Wisconsin-Madison, 1101 University Ave, Madison, WI 53706, USA; b Department of Chemistry, University of Johannesburg, Auckland Park Kingsway Campus, Johannesburg 2006, South Africa

## Abstract

In the title compound, [Au_2_(C_6_H_8_N_2_S_4_)(C_18_H_15_P)_4_]·2CHCl_3_, the digold complex resides on a crystallographic inversion center and co-crystallizes with two mol­ecules of chloro­form solvent. The piperazine-1,4-dicarbodithio­ate linker has an almost ideal chair conformation. The geometry about the gold atoms is severely distorted tetra­hedral punctuated by a very acute S—Au—S bite angle.

## Related literature

For stabilization of gold salts by dithio­carbonates, see: Fernandez *et al.* (1998 ▶). For use of piperazine dithio­carbamates as ligands used to engineer multimetallic assemblies, see: Wilton-Ely *et al.* (2008 ▶); Knight *et al.* (2009*a* ▶,*b* ▶); Oliver *et al.* (2011 ▶). For the copper analgoue, see: Kumar *et al.* (2009 ▶). For other related gold complexes, see: Razak *et al.* (2000 ▶); Jian *et al.* (2000 ▶). A mol­ecular geometry check was performed with *Mogul*, see: Bruno *et al.* (2002 ▶). Related compounds were found in the Cambridge Structural Database (Allen, 2002 ▶). For ring analysis, see: Cremer & Pople (1975 ▶).
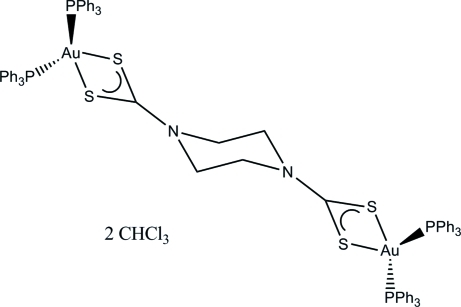


## Experimental

### 

#### Crystal data

[Au_2_(C_6_H_8_N_2_S_4_)(C_18_H_15_P)_4_]·2CHCl_3_
*M* *_r_* = 1918.13
Triclinic, 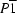

*a* = 12.8455 (17) Å
*b* = 13.2879 (10) Å
*c* = 13.4197 (9) Å
α = 119.572 (2)°
β = 101.544 (2)°
γ = 96.039 (2)°
*V* = 1895.2 (3) Å^3^
*Z* = 1
Cu *K*α radiation
μ = 11.30 mm^−1^
*T* = 100 K
0.44 × 0.35 × 0.29 mm

#### Data collection

Bruker SMART APEXII diffractometer
Absorption correction: multi-scan (*SADABS*; Bruker, 2007 ▶) *T*_min_ = 0.083, *T*_max_ = 0.140
30338 measured reflections
7089 independent reflections
7080 reflections with *I* > 2σ(*I*)
*R*_int_ = 0.031

#### Refinement

*R*[*F* ^2^ > 2σ(*F* ^2^)] = 0.028
*wR*(*F* ^2^) = 0.074
*S* = 1.15
7089 reflections
442 parameters
H-atom parameters constrained
Δρ_max_ = 2.40 e Å^−3^
Δρ_min_ = −1.35 e Å^−3^

### 

Data collection: *APEX2* (Bruker, 2007 ▶); cell refinement: *SAINT* (Bruker, 2007 ▶); data reduction: *SAINT*; program(s) used to solve structure: *SHELXS97* (Sheldrick, 2008 ▶); program(s) used to refine structure: *SHELXTL* (Sheldrick, 2008 ▶), *FCF_filter* (Guzei, 2007 ▶) and *INSerter* (Guzei, 2007 ▶); molecular graphics: *SHELXTL* and *DIAMOND* (Brandenburg, 1999 ▶); software used to prepare material for publication: *SHELXTL*, *publCIF* (Westrip, 2010 ▶) and *modiCIFer* (Guzei, 2007 ▶).

## Supplementary Material

Crystal structure: contains datablock(s) global, I. DOI: 10.1107/S1600536811044229/ng5253sup1.cif

Structure factors: contains datablock(s) I. DOI: 10.1107/S1600536811044229/ng5253Isup2.hkl

Additional supplementary materials:  crystallographic information; 3D view; checkCIF report

## Figures and Tables

**Table:** 

Au1—P2	2.2994 (8)
Au1—P1	2.3233 (8)
Au1—S2	2.6133 (8)
Au1—S1	2.7414 (8)

**Table:** 

P2—Au1—P1	134.65 (3)
P2—Au1—S2	116.81 (3)
P1—Au1—S2	107.10 (3)
P2—Au1—S1	107.34 (3)
P1—Au1—S1	99.39 (3)
S2—Au1—S1	67.03 (2)

**Fig. 1.**
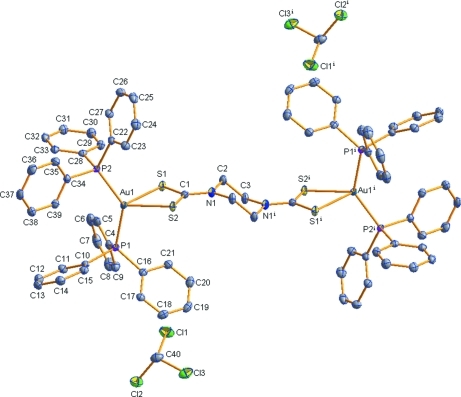
Molecular structure of (I) (Brandenburg, 1999). The thermal ellipsoids are shown at 50% probability level. All hydrogen atoms were omitted. Symmetry code: (i) -x,2-y, 2-z.

## supplementary crystallographic information

### Comment

Dithiocarbamates have long been used as ligands to stabilize gold(I) and gold(III) salts (Fernandez *et al.*, 1998), but piperazine dithiocarbamates are currently receiving a lot more attention as ligands that can be used to engineer multimetallic assemblies including making gold nanoparticles (Wilton-Ely *et al.*, 2008; Knight *et al.* 2009*a*; Knight *et al.*, 2009*b*; Oliver *et al.*, 2011). We recently isolated the title compound (I) *via* a slight modification of one of the routes described in the aforementioned literature.

The crystal structure of (I) contains the digold complex residing on a crystallographic inversion center and two molecules of solvent chloroform solvent per digold complex. The piperzine dithiocarbamate linker exhibits an almost ideal chair conformation (puckering coordinates θ=177.97 (1)° φ=0°, Cremer & Pople, 1975) similar to the analogous compounds with group ten square-planar metal centers nickel, palladium, and platinum (Knight *et al.*, 2009*a*) and the tetrahedral copper analogue (Kumar *et al.*, 2009). All bond distances and angles are typical as confirmed by a *Mogul* geometry check except for the S1—C1—S2, S1—C1—N1, and S2—C1—N1 angles (Bruno *et al.*, 2002). However these angles in (I) are similar to those in the closely related compounds (*N*,*N*-diisopropyldithiocarbamato-*S*,*S*')-bis(triphenylphosphane-P)-gold(I) (Jian *et al.*, 2000) and (piperidine-1-carbodithioato-*S*,*S*')-bis(triphenylphosphane-P)-gold(I) (Razak *et al.*, 2000).

The geometry about the gold atom is severely distorted tetrahedral with the dihedral angle between the planes defined by atoms S1, Au1, S1 and P1, Au1, P1 measuring 88.77 (3)°. Such a distorted tetrahedral geometry and acute S—Au—S bite angle (67.03 (2)°) are typical of complexes where gold is bonded to two phosphorous atoms and two sulfur atoms of a bidentale ligand forming a four-membered metallocycle. For eight such compounds in the Cambridge Structural Database (CSD; August 2011 update; Allen, 2002) the S—Au—S bite angle has an average of 66 (3)°. These compounds also have very large P—Au—P angles with a 135 (5)° average corresponding well to the 134.65 (3)° value found in (I). The copper analogue of (I) also exhibits a similarly distorted tetrahedral geometry but with a larger S—Cu—S bite angle of 75.41 (2)° (Kumar *et al.*, 2009). The group ten analogues exhibit distorted square planar geometries with larger S—metal—S bite angles that average 77 (3)° (Knight *et al.*, 2009*a*). The Au—S distances in (I) differ by 0.1281 Å; this value agrees well with the differences in the two Au—S bonds in the eight related compounds in the CSD where such distances differ by an average of 0.18 (11) Å.

### Experimental

To a solution of potassium piperazine-1,4-bis(dithiocarbamate) (0.17 g, 0.57 mmol) in water (10 mL) was added a solution of [AuCl(PPh_3_)] (0.40 g, 0.81 mmol) in dichloromethane (10 mL). The biphasic reaction mixture was stirred for 30 minutes. The organic layer was separated and layered with chloroform and hexane to yield a yellow solid. Yield: 0.32 g (69%). ^1^H NMR (CDCl_3_): δ 7.53 (m, 12H), 7.42 (m, 18H), 4.30 (s, 8H). ^13^C{^1^H} NMR (CDCl_3_): δ 208.2 (2 C, C=S), 134.1, 130.7, 128.9, 50.1. ^31^ P{^1^H} NMR (CDCl_3_): δ 28.9. ESI-MS (m/*z*): 1155 ([*M*], 5%), 721 [Au(PPh_3_)_2_]^+^, 100%). IR (ATR, cm^-1^): υ(_C—N_) = 1451, υ(_C=S_) = 1026, υ(_C—S_) = 997. Anal. Calc. for C_78_H_68_Au_2_N_2_P_2_S_4_^.^CHCl_3_: C 50.09, H 3.68, N 1.46. Found: C 49.73, H 3.76, N 1.60%.

### Refinement

All H-atoms attached to carbon atoms were placed in idealized locations and refined as riding with appropriate thermal displacement coefficients *U*_iso_(H) = 1.2 times *U*_eq_(bearing atom). Default effective *X*—H distances for T = -173.0°C C(*sp* 3)–1H=1.00, C(*sp* 3)–2H=0.99, C(*sp* 2)–H=0.95. The final difference map had a peak and a hole in the vicinities of Au.

### Crystal data

**Table:**

[Au_2_(C_6_H_8_N_2_S_4_)(C_18_H_15_P)_4_]·2CHCl_3_	*Z* = 1
*M**_r_* = 1918.13	*F*(000) = 948
Triclinic, *P*1	*D*_x_ = 1.681 Mg m^−^^3^
Hall symbol: -P 1	Cu *K*α radiation, λ = 1.54178 Å
*a* = 12.8455 (17) Å	Cell parameters from 9936 reflections
*b* = 13.2879 (10) Å	θ = 3.6–71.7°
*c* = 13.4197 (9) Å	µ = 11.30 mm^−^^1^
α = 119.572 (2)°	*T* = 100 K
β = 101.544 (2)°	Block, colourless
γ = 96.039 (2)°	0.44 × 0.35 × 0.29 mm
*V* = 1895.2 (3) Å^3^	

### Data collection

**Table:**

Bruker SMART APEXII diffractometer	7089 independent reflections
Radiation source: fine-focus sealed tube	7080 reflections with *I* > 2σ(*I*)
graphite	*R*_int_ = 0.031
0.50° ω and 0.5 ° φ scans	θ_max_ = 72.3°, θ_min_ = 3.6°
Absorption correction: multi-scan (*SADABS*; Bruker, 2007)	*h* = −15→14
*T*_min_ = 0.083, *T*_max_ = 0.140	*k* = −16→16
30338 measured reflections	*l* = −16→16

### Refinement

**Table:**

Refinement on *F*^2^	Primary atom site location: structure-invariant direct methods
Least-squares matrix: full	Secondary atom site location: difference Fourier map
*R*[*F*^2^ > 2σ(*F*^2^)] = 0.028	Hydrogen site location: inferred from neighbouring sites
*wR*(*F*^2^) = 0.074	H-atom parameters constrained
*S* = 1.15	*w* = 1/[σ^2^(*F*_o_^2^) + (0.0416*P*)^2^ + 3.5159*P*] where *P* = (*F*_o_^2^ + 2*F*_c_^2^)/3
7089 reflections	(Δ/σ)_max_ = 0.001
442 parameters	Δρ_max_ = 2.40 e Å^−^^3^
0 restraints	Δρ_min_ = −1.35 e Å^−^^3^

### Special details

**Table:**

Geometry. All e.s.d.'s (except the e.s.d. in the dihedral angle between two l.s. planes) are estimated using the full covariance matrix. The cell e.s.d.'s are taken into account individually in the estimation of e.s.d.'s in distances, angles and torsion angles; correlations between e.s.d.'s in cell parameters are only used when they are defined by crystal symmetry. An approximate (isotropic) treatment of cell e.s.d.'s is used for estimating e.s.d.'s involving l.s. planes.
Refinement. Refinement of *F*^2^ against ALL reflections. The weighted *R*-factor *wR* and goodness of fit *S* are based on *F*^2^, conventional *R*-factors *R* are based on *F*, with *F* set to zero for negative *F*^2^. The threshold expression of *F*^2^ > σ(*F*^2^) is used only for calculating *R*-factors(gt) *etc*. and is not relevant to the choice of reflections for refinement. *R*-factors based on *F*^2^ are statistically about twice as large as those based on *F*, and *R*- factors based on ALL data will be even larger.

### Fractional atomic coordinates and isotropic or equivalent isotropic displacement parameters (Å^2^)

**Table:**

	*x*	*y*	*z*	*U*_iso_*/*U*_eq_	
Au1	0.234436 (10)	0.776500 (10)	0.653197 (10)	0.01426 (6)	
S1	0.25387 (6)	0.94442 (7)	0.88645 (7)	0.01770 (16)	
S2	0.04873 (6)	0.82819 (7)	0.68645 (7)	0.01689 (16)	
P1	0.24564 (7)	0.61270 (7)	0.67150 (7)	0.01428 (16)	
P2	0.32654 (7)	0.86933 (7)	0.57904 (7)	0.01350 (16)	
N1	0.0585 (2)	0.9761 (2)	0.9150 (2)	0.0168 (5)	
C1	0.1155 (3)	0.9217 (3)	0.8365 (3)	0.0156 (6)	
C2	0.1115 (3)	1.0645 (3)	1.0418 (3)	0.0195 (7)	
H2AB	0.1051	1.1446	1.0584	0.023*	
H2AA	0.1903	1.0655	1.0614	0.023*	
C3	0.0590 (3)	1.0359 (3)	1.1199 (3)	0.0192 (7)	
H3AA	0.0737	0.9608	1.1113	0.023*	
H3AB	0.0913	1.1005	1.2050	0.023*	
C4	0.3628 (3)	0.6456 (3)	0.7941 (3)	0.0183 (7)	
C5	0.4568 (3)	0.7301 (3)	0.8254 (3)	0.0215 (7)	
H5AA	0.4581	0.7723	0.7854	0.026*	
C6	0.5488 (3)	0.7527 (4)	0.9152 (3)	0.0285 (8)	
H6AA	0.6130	0.8098	0.9357	0.034*	
C7	0.5471 (3)	0.6927 (4)	0.9744 (3)	0.0311 (9)	
H7AA	0.6101	0.7085	1.0357	0.037*	
C8	0.4537 (3)	0.6093 (4)	0.9447 (3)	0.0301 (8)	
H8AA	0.4526	0.5687	0.9863	0.036*	
C9	0.3620 (3)	0.5849 (3)	0.8550 (3)	0.0238 (7)	
H9AA	0.2985	0.5270	0.8346	0.029*	
C10	0.2675 (3)	0.4871 (3)	0.5424 (3)	0.0159 (6)	
C11	0.3632 (3)	0.4487 (3)	0.5474 (3)	0.0203 (7)	
H11A	0.4171	0.4825	0.6223	0.024*	
C12	0.3813 (3)	0.3601 (3)	0.4429 (3)	0.0245 (7)	
H12A	0.4475	0.3346	0.4470	0.029*	
C13	0.3025 (3)	0.3101 (3)	0.3339 (3)	0.0229 (7)	
H13A	0.3145	0.2498	0.2631	0.027*	
C14	0.2067 (3)	0.3477 (3)	0.3280 (3)	0.0215 (7)	
H14A	0.1527	0.3128	0.2530	0.026*	
C15	0.1887 (3)	0.4362 (3)	0.4311 (3)	0.0190 (7)	
H15A	0.1228	0.4624	0.4262	0.023*	
C16	0.1298 (3)	0.5531 (3)	0.7022 (3)	0.0168 (6)	
C17	0.0658 (3)	0.4376 (3)	0.6282 (3)	0.0187 (7)	
H17A	0.0823	0.3836	0.5574	0.022*	
C18	−0.0229 (3)	0.4005 (3)	0.6578 (3)	0.0236 (7)	
H18A	−0.0670	0.3215	0.6065	0.028*	
C19	−0.0467 (3)	0.4788 (3)	0.7617 (3)	0.0243 (7)	
H19A	−0.1067	0.4534	0.7820	0.029*	
C20	0.0174 (3)	0.5941 (3)	0.8355 (3)	0.0248 (7)	
H20A	0.0017	0.6474	0.9072	0.030*	
C21	0.1042 (3)	0.6322 (3)	0.8060 (3)	0.0204 (7)	
H21A	0.1464	0.7121	0.8561	0.025*	
C22	0.2798 (3)	0.9894 (3)	0.5672 (3)	0.0169 (6)	
C23	0.1719 (3)	0.9678 (3)	0.5049 (3)	0.0218 (7)	
H23A	0.1233	0.8929	0.4726	0.026*	
C24	0.1338 (3)	1.0550 (3)	0.4894 (3)	0.0269 (8)	
H24A	0.0604	1.0382	0.4438	0.032*	
C25	0.2025 (3)	1.1658 (3)	0.5399 (3)	0.0262 (8)	
H25A	0.1764	1.2252	0.5293	0.031*	
C26	0.3099 (3)	1.1904 (3)	0.6063 (3)	0.0242 (7)	
H26A	0.3567	1.2672	0.6431	0.029*	
C27	0.3491 (3)	1.1018 (3)	0.6189 (3)	0.0207 (7)	
H27A	0.4230	1.1181	0.6628	0.025*	
C28	0.4678 (3)	0.9384 (3)	0.6736 (3)	0.0157 (6)	
C29	0.4856 (3)	1.0177 (3)	0.7967 (3)	0.0187 (7)	
H29A	0.4249	1.0341	0.8276	0.022*	
C30	0.5910 (3)	1.0720 (3)	0.8732 (3)	0.0214 (7)	
H30A	0.6027	1.1262	0.9563	0.026*	
C31	0.6797 (3)	1.0473 (3)	0.8284 (3)	0.0208 (7)	
H31A	0.7520	1.0844	0.8812	0.025*	
C32	0.6637 (3)	0.9691 (3)	0.7077 (3)	0.0206 (7)	
H32A	0.7247	0.9524	0.6776	0.025*	
C33	0.5574 (3)	0.9145 (3)	0.6298 (3)	0.0177 (6)	
H33A	0.5464	0.8610	0.5467	0.021*	
C34	0.3386 (3)	0.7666 (3)	0.4312 (3)	0.0154 (6)	
C35	0.3435 (3)	0.7995 (3)	0.3476 (3)	0.0205 (7)	
H35A	0.3372	0.8769	0.3657	0.025*	
C36	0.3575 (3)	0.7189 (3)	0.2381 (3)	0.0247 (7)	
H36A	0.3597	0.7410	0.1810	0.030*	
C37	0.3683 (3)	0.6067 (3)	0.2119 (3)	0.0220 (7)	
H37A	0.3790	0.5524	0.1374	0.026*	
C38	0.3635 (3)	0.5729 (3)	0.2941 (3)	0.0220 (7)	
H38A	0.3715	0.4960	0.2763	0.026*	
C39	0.3470 (3)	0.6526 (3)	0.4026 (3)	0.0184 (7)	
H39A	0.3413	0.6288	0.4578	0.022*	
Cl1	0.15739 (10)	0.41053 (9)	0.95175 (11)	0.0451 (3)	
Cl2	0.14677 (11)	0.15846 (10)	0.81701 (11)	0.0455 (3)	
Cl3	−0.05265 (9)	0.24127 (10)	0.82114 (9)	0.0383 (2)	
C40	0.0848 (3)	0.2721 (4)	0.8232 (4)	0.0305 (8)	
H40A	0.0863	0.2762	0.7511	0.037*	

### Atomic displacement parameters (Å^2^)

**Table:**

	*U*^11^	*U*^22^	*U*^33^	*U*^12^	*U*^13^	*U*^23^
Au1	0.01735 (9)	0.01435 (8)	0.01405 (8)	0.00500 (5)	0.00798 (6)	0.00817 (6)
S1	0.0143 (4)	0.0222 (4)	0.0133 (3)	0.0047 (3)	0.0051 (3)	0.0065 (3)
S2	0.0149 (4)	0.0209 (4)	0.0123 (3)	0.0050 (3)	0.0044 (3)	0.0066 (3)
P1	0.0157 (4)	0.0147 (4)	0.0139 (4)	0.0043 (3)	0.0058 (3)	0.0080 (3)
P2	0.0156 (4)	0.0141 (4)	0.0136 (4)	0.0045 (3)	0.0063 (3)	0.0084 (3)
N1	0.0135 (14)	0.0212 (14)	0.0138 (13)	0.0050 (11)	0.0049 (10)	0.0073 (11)
C1	0.0168 (16)	0.0153 (15)	0.0155 (15)	0.0025 (12)	0.0057 (12)	0.0085 (13)
C2	0.0175 (17)	0.0210 (16)	0.0151 (16)	0.0036 (13)	0.0056 (13)	0.0058 (14)
C3	0.0156 (17)	0.0260 (17)	0.0147 (15)	0.0079 (13)	0.0062 (12)	0.0086 (14)
C4	0.0197 (17)	0.0199 (16)	0.0145 (15)	0.0068 (13)	0.0062 (13)	0.0077 (13)
C5	0.0224 (18)	0.0199 (16)	0.0170 (16)	0.0042 (13)	0.0077 (13)	0.0054 (14)
C6	0.0201 (19)	0.0315 (19)	0.0225 (18)	0.0079 (15)	0.0067 (14)	0.0057 (16)
C7	0.028 (2)	0.041 (2)	0.0168 (17)	0.0185 (17)	0.0045 (15)	0.0091 (16)
C8	0.035 (2)	0.043 (2)	0.0243 (19)	0.0203 (18)	0.0115 (16)	0.0225 (18)
C9	0.027 (2)	0.0289 (18)	0.0237 (18)	0.0109 (15)	0.0102 (15)	0.0175 (16)
C10	0.0218 (18)	0.0128 (14)	0.0159 (15)	0.0054 (12)	0.0090 (13)	0.0080 (12)
C11	0.0209 (18)	0.0190 (16)	0.0211 (17)	0.0069 (13)	0.0052 (14)	0.0105 (14)
C12	0.0243 (19)	0.0223 (17)	0.0285 (19)	0.0087 (14)	0.0117 (15)	0.0124 (15)
C13	0.030 (2)	0.0157 (15)	0.0219 (17)	0.0062 (14)	0.0130 (15)	0.0071 (14)
C14	0.0243 (19)	0.0198 (16)	0.0166 (16)	0.0027 (14)	0.0037 (13)	0.0084 (14)
C15	0.0199 (18)	0.0184 (16)	0.0200 (16)	0.0053 (13)	0.0060 (13)	0.0108 (14)
C16	0.0155 (17)	0.0200 (16)	0.0204 (16)	0.0062 (13)	0.0053 (12)	0.0143 (14)
C17	0.0193 (18)	0.0195 (16)	0.0206 (16)	0.0062 (13)	0.0061 (13)	0.0125 (14)
C18	0.0196 (18)	0.0260 (18)	0.0286 (19)	0.0013 (14)	0.0043 (14)	0.0185 (16)
C19	0.0173 (18)	0.037 (2)	0.0321 (19)	0.0067 (15)	0.0089 (14)	0.0270 (17)
C20	0.026 (2)	0.0334 (19)	0.0237 (18)	0.0115 (15)	0.0123 (15)	0.0189 (16)
C21	0.0233 (18)	0.0206 (16)	0.0202 (16)	0.0052 (13)	0.0079 (13)	0.0122 (14)
C22	0.0216 (18)	0.0187 (15)	0.0169 (15)	0.0095 (13)	0.0105 (13)	0.0113 (13)
C23	0.0222 (18)	0.0218 (17)	0.0239 (17)	0.0069 (14)	0.0073 (14)	0.0132 (15)
C24	0.027 (2)	0.032 (2)	0.0281 (19)	0.0129 (16)	0.0090 (15)	0.0191 (17)
C25	0.032 (2)	0.0299 (19)	0.0308 (19)	0.0190 (16)	0.0162 (16)	0.0216 (17)
C26	0.030 (2)	0.0188 (16)	0.0276 (18)	0.0067 (14)	0.0125 (15)	0.0134 (15)
C27	0.0203 (18)	0.0218 (17)	0.0244 (17)	0.0077 (14)	0.0080 (14)	0.0142 (15)
C28	0.0176 (17)	0.0152 (14)	0.0173 (15)	0.0045 (12)	0.0047 (12)	0.0108 (13)
C29	0.0180 (17)	0.0222 (16)	0.0182 (16)	0.0062 (13)	0.0068 (13)	0.0114 (14)
C30	0.0228 (18)	0.0239 (17)	0.0165 (16)	0.0060 (14)	0.0044 (13)	0.0104 (14)
C31	0.0158 (17)	0.0238 (17)	0.0240 (17)	0.0056 (13)	0.0027 (13)	0.0143 (15)
C32	0.0203 (18)	0.0236 (17)	0.0252 (18)	0.0114 (14)	0.0110 (14)	0.0154 (15)
C33	0.0199 (17)	0.0192 (15)	0.0199 (16)	0.0070 (13)	0.0080 (13)	0.0132 (14)
C34	0.0129 (16)	0.0172 (15)	0.0152 (15)	0.0041 (12)	0.0048 (12)	0.0075 (13)
C35	0.0275 (19)	0.0193 (16)	0.0176 (16)	0.0062 (13)	0.0086 (14)	0.0109 (14)
C36	0.031 (2)	0.0309 (19)	0.0186 (17)	0.0078 (15)	0.0111 (14)	0.0162 (15)
C37	0.0231 (18)	0.0237 (17)	0.0152 (16)	0.0053 (14)	0.0097 (13)	0.0059 (14)
C38	0.0239 (19)	0.0192 (16)	0.0224 (17)	0.0064 (13)	0.0102 (14)	0.0093 (14)
C39	0.0196 (17)	0.0208 (16)	0.0169 (15)	0.0051 (13)	0.0068 (13)	0.0109 (14)
Cl1	0.0397 (6)	0.0299 (5)	0.0451 (6)	−0.0012 (4)	0.0001 (5)	0.0112 (5)
Cl2	0.0608 (7)	0.0342 (5)	0.0547 (7)	0.0180 (5)	0.0278 (6)	0.0276 (5)
Cl3	0.0355 (5)	0.0420 (5)	0.0346 (5)	−0.0020 (4)	0.0028 (4)	0.0230 (4)
C40	0.035 (2)	0.030 (2)	0.0264 (19)	0.0009 (16)	0.0048 (16)	0.0183 (17)

### Geometric parameters (Å, °)

**Table:**

Au1—P2	2.2994 (8)	C17—H17A	0.9500
Au1—P1	2.3233 (8)	C18—C19	1.388 (6)
Au1—S2	2.6133 (8)	C18—H18A	0.9500
Au1—S1	2.7414 (8)	C19—C20	1.384 (5)
S1—C1	1.706 (3)	C19—H19A	0.9500
S2—C1	1.718 (3)	C20—C21	1.383 (5)
P1—C10	1.818 (3)	C20—H20A	0.9500
P1—C4	1.823 (4)	C21—H21A	0.9500
P1—C16	1.825 (3)	C22—C23	1.386 (5)
P2—C34	1.822 (3)	C22—C27	1.398 (5)
P2—C28	1.823 (3)	C23—C24	1.393 (5)
P2—C22	1.829 (3)	C23—H23A	0.9500
N1—C1	1.354 (4)	C24—C25	1.382 (6)
N1—C3^i^	1.454 (4)	C24—H24A	0.9500
N1—C2	1.460 (4)	C25—C26	1.389 (6)
C2—C3	1.524 (5)	C25—H25A	0.9500
C2—H2AB	0.9900	C26—C27	1.397 (5)
C2—H2AA	0.9900	C26—H26A	0.9500
C3—N1^i^	1.454 (4)	C27—H27A	0.9500
C3—H3AA	0.9900	C28—C33	1.392 (5)
C3—H3AB	0.9900	C28—C29	1.403 (5)
C4—C5	1.393 (5)	C29—C30	1.385 (5)
C4—C9	1.405 (5)	C29—H29A	0.9500
C5—C6	1.392 (5)	C30—C31	1.387 (5)
C5—H5AA	0.9500	C30—H30A	0.9500
C6—C7	1.378 (6)	C31—C32	1.381 (5)
C6—H6AA	0.9500	C31—H31A	0.9500
C7—C8	1.386 (6)	C32—C33	1.397 (5)
C7—H7AA	0.9500	C32—H32A	0.9500
C8—C9	1.380 (5)	C33—H33A	0.9500
C8—H8AA	0.9500	C34—C39	1.390 (5)
C9—H9AA	0.9500	C34—C35	1.400 (5)
C10—C11	1.381 (5)	C35—C36	1.390 (5)
C10—C15	1.403 (5)	C35—H35A	0.9500
C11—C12	1.402 (5)	C36—C37	1.383 (5)
C11—H11A	0.9500	C36—H36A	0.9500
C12—C13	1.382 (5)	C37—C38	1.389 (5)
C12—H12A	0.9500	C37—H37A	0.9500
C13—C14	1.378 (5)	C38—C39	1.392 (5)
C13—H13A	0.9500	C38—H38A	0.9500
C14—C15	1.390 (5)	C39—H39A	0.9500
C14—H14A	0.9500	Cl1—C40	1.758 (4)
C15—H15A	0.9500	Cl2—C40	1.754 (4)
C16—C17	1.386 (5)	Cl3—C40	1.762 (4)
C16—C21	1.402 (5)	C40—H40A	1.0000
C17—C18	1.398 (5)		
			
P2—Au1—P1	134.65 (3)	C21—C16—P1	116.5 (3)
P2—Au1—S2	116.81 (3)	C16—C17—C18	120.1 (3)
P1—Au1—S2	107.10 (3)	C16—C17—H17A	120.0
P2—Au1—S1	107.34 (3)	C18—C17—H17A	120.0
P1—Au1—S1	99.39 (3)	C19—C18—C17	120.2 (3)
S2—Au1—S1	67.03 (2)	C19—C18—H18A	119.9
C1—S1—Au1	84.64 (11)	C17—C18—H18A	119.9
C1—S2—Au1	88.55 (12)	C20—C19—C18	119.6 (3)
C10—P1—C4	103.04 (16)	C20—C19—H19A	120.2
C10—P1—C16	106.19 (15)	C18—C19—H19A	120.2
C4—P1—C16	103.85 (15)	C21—C20—C19	120.6 (3)
C10—P1—Au1	113.94 (10)	C21—C20—H20A	119.7
C4—P1—Au1	112.18 (11)	C19—C20—H20A	119.7
C16—P1—Au1	116.33 (11)	C20—C21—C16	120.1 (3)
C34—P2—C28	104.10 (15)	C20—C21—H21A	120.0
C34—P2—C22	104.72 (15)	C16—C21—H21A	120.0
C28—P2—C22	103.69 (15)	C23—C22—C27	119.1 (3)
C34—P2—Au1	113.39 (11)	C23—C22—P2	118.6 (3)
C28—P2—Au1	109.59 (10)	C27—C22—P2	122.3 (3)
C22—P2—Au1	119.85 (11)	C22—C23—C24	120.6 (3)
C1—N1—C3^i^	123.8 (3)	C22—C23—H23A	119.7
C1—N1—C2	122.7 (3)	C24—C23—H23A	119.7
C3^i^—N1—C2	113.0 (3)	C25—C24—C23	120.2 (4)
N1—C1—S1	120.2 (2)	C25—C24—H24A	119.9
N1—C1—S2	120.3 (3)	C23—C24—H24A	119.9
S1—C1—S2	119.56 (19)	C24—C25—C26	119.9 (3)
N1—C2—C3	110.7 (3)	C24—C25—H25A	120.1
N1—C2—H2AB	109.5	C26—C25—H25A	120.1
C3—C2—H2AB	109.5	C25—C26—C27	119.9 (3)
N1—C2—H2AA	109.5	C25—C26—H26A	120.0
C3—C2—H2AA	109.5	C27—C26—H26A	120.0
H2AB—C2—H2AA	108.1	C26—C27—C22	120.2 (3)
N1^i^—C3—C2	110.2 (3)	C26—C27—H27A	119.9
N1^i^—C3—H3AA	109.6	C22—C27—H27A	119.9
C2—C3—H3AA	109.6	C33—C28—C29	119.1 (3)
N1^i^—C3—H3AB	109.6	C33—C28—P2	123.2 (3)
C2—C3—H3AB	109.6	C29—C28—P2	117.7 (3)
H3AA—C3—H3AB	108.1	C30—C29—C28	120.4 (3)
C5—C4—C9	119.1 (3)	C30—C29—H29A	119.8
C5—C4—P1	119.3 (3)	C28—C29—H29A	119.8
C9—C4—P1	121.6 (3)	C29—C30—C31	119.9 (3)
C6—C5—C4	120.2 (3)	C29—C30—H30A	120.0
C6—C5—H5AA	119.9	C31—C30—H30A	120.0
C4—C5—H5AA	119.9	C32—C31—C30	120.5 (3)
C7—C6—C5	120.2 (4)	C32—C31—H31A	119.8
C7—C6—H6AA	119.9	C30—C31—H31A	119.8
C5—C6—H6AA	119.9	C31—C32—C33	119.9 (3)
C6—C7—C8	120.0 (4)	C31—C32—H32A	120.0
C6—C7—H7AA	120.0	C33—C32—H32A	120.0
C8—C7—H7AA	120.0	C28—C33—C32	120.2 (3)
C9—C8—C7	120.5 (4)	C28—C33—H33A	119.9
C9—C8—H8AA	119.8	C32—C33—H33A	119.9
C7—C8—H8AA	119.8	C39—C34—C35	119.2 (3)
C8—C9—C4	119.9 (4)	C39—C34—P2	118.2 (2)
C8—C9—H9AA	120.0	C35—C34—P2	122.6 (2)
C4—C9—H9AA	120.0	C36—C35—C34	120.0 (3)
C11—C10—C15	119.0 (3)	C36—C35—H35A	120.0
C11—C10—P1	122.6 (3)	C34—C35—H35A	120.0
C15—C10—P1	118.0 (3)	C37—C36—C35	120.2 (3)
C10—C11—C12	120.6 (3)	C37—C36—H36A	119.9
C10—C11—H11A	119.7	C35—C36—H36A	119.9
C12—C11—H11A	119.7	C36—C37—C38	120.3 (3)
C13—C12—C11	119.8 (3)	C36—C37—H37A	119.8
C13—C12—H12A	120.1	C38—C37—H37A	119.8
C11—C12—H12A	120.1	C37—C38—C39	119.5 (3)
C14—C13—C12	120.1 (3)	C37—C38—H38A	120.2
C14—C13—H13A	120.0	C39—C38—H38A	120.2
C12—C13—H13A	120.0	C34—C39—C38	120.7 (3)
C13—C14—C15	120.4 (3)	C34—C39—H39A	119.6
C13—C14—H14A	119.8	C38—C39—H39A	119.6
C15—C14—H14A	119.8	Cl2—C40—Cl1	110.6 (2)
C14—C15—C10	120.1 (3)	Cl2—C40—Cl3	110.7 (2)
C14—C15—H15A	120.0	Cl1—C40—Cl3	110.3 (2)
C10—C15—H15A	120.0	Cl2—C40—H40A	108.4
C17—C16—C21	119.4 (3)	Cl1—C40—H40A	108.4
C17—C16—P1	124.1 (3)	Cl3—C40—H40A	108.4
			
P2—Au1—S1—C1	115.18 (11)	C13—C14—C15—C10	−0.8 (5)
P1—Au1—S1—C1	−101.89 (11)	C11—C10—C15—C14	0.7 (5)
S2—Au1—S1—C1	2.79 (11)	P1—C10—C15—C14	173.9 (3)
P2—Au1—S2—C1	−101.30 (11)	C10—P1—C16—C17	−7.7 (3)
P1—Au1—S2—C1	90.39 (11)	C4—P1—C16—C17	−116.0 (3)
S1—Au1—S2—C1	−2.76 (11)	Au1—P1—C16—C17	120.2 (3)
P2—Au1—P1—C10	−40.63 (13)	C10—P1—C16—C21	174.0 (3)
S2—Au1—P1—C10	124.64 (13)	C4—P1—C16—C21	65.7 (3)
S1—Au1—P1—C10	−166.64 (13)	Au1—P1—C16—C21	−58.0 (3)
P2—Au1—P1—C4	75.96 (12)	C21—C16—C17—C18	−0.4 (5)
S2—Au1—P1—C4	−118.77 (12)	P1—C16—C17—C18	−178.6 (3)
S1—Au1—P1—C4	−50.05 (12)	C16—C17—C18—C19	−0.6 (5)
P2—Au1—P1—C16	−164.70 (12)	C17—C18—C19—C20	0.4 (5)
S2—Au1—P1—C16	0.57 (13)	C18—C19—C20—C21	0.7 (5)
S1—Au1—P1—C16	69.29 (13)	C19—C20—C21—C16	−1.7 (5)
P1—Au1—P2—C34	48.64 (13)	C17—C16—C21—C20	1.6 (5)
S2—Au1—P2—C34	−115.56 (12)	P1—C16—C21—C20	179.9 (3)
S1—Au1—P2—C34	171.92 (12)	C34—P2—C22—C23	74.3 (3)
P1—Au1—P2—C28	−67.20 (12)	C28—P2—C22—C23	−176.8 (3)
S2—Au1—P2—C28	128.61 (11)	Au1—P2—C22—C23	−54.3 (3)
S1—Au1—P2—C28	56.08 (11)	C34—P2—C22—C27	−105.8 (3)
P1—Au1—P2—C22	173.21 (13)	C28—P2—C22—C27	3.1 (3)
S2—Au1—P2—C22	9.01 (13)	Au1—P2—C22—C27	125.6 (3)
S1—Au1—P2—C22	−63.52 (13)	C27—C22—C23—C24	2.8 (5)
C3^i^—N1—C1—S1	178.5 (2)	P2—C22—C23—C24	−177.3 (3)
C2—N1—C1—S1	6.3 (4)	C22—C23—C24—C25	−2.4 (6)
C3^i^—N1—C1—S2	−2.2 (4)	C23—C24—C25—C26	0.1 (6)
C2—N1—C1—S2	−174.4 (2)	C24—C25—C26—C27	1.8 (5)
Au1—S1—C1—N1	174.8 (3)	C25—C26—C27—C22	−1.5 (5)
Au1—S1—C1—S2	−4.50 (17)	C23—C22—C27—C26	−0.8 (5)
Au1—S2—C1—N1	−174.6 (3)	P2—C22—C27—C26	179.3 (3)
Au1—S2—C1—S1	4.70 (18)	C34—P2—C28—C33	2.9 (3)
C1—N1—C2—C3	−131.2 (3)	C22—P2—C28—C33	−106.4 (3)
C3^i^—N1—C2—C3	55.8 (4)	Au1—P2—C28—C33	124.5 (2)
N1—C2—C3—N1^i^	−54.2 (4)	C34—P2—C28—C29	−175.9 (2)
C10—P1—C4—C5	92.7 (3)	C22—P2—C28—C29	74.8 (3)
C16—P1—C4—C5	−156.7 (3)	Au1—P2—C28—C29	−54.3 (3)
Au1—P1—C4—C5	−30.2 (3)	C33—C28—C29—C30	0.5 (5)
C10—P1—C4—C9	−85.0 (3)	P2—C28—C29—C30	179.4 (3)
C16—P1—C4—C9	25.6 (3)	C28—C29—C30—C31	−0.6 (5)
Au1—P1—C4—C9	152.0 (3)	C29—C30—C31—C32	0.3 (5)
C9—C4—C5—C6	0.6 (5)	C30—C31—C32—C33	0.1 (5)
P1—C4—C5—C6	−177.2 (3)	C29—C28—C33—C32	−0.1 (5)
C4—C5—C6—C7	−0.7 (5)	P2—C28—C33—C32	−178.9 (2)
C5—C6—C7—C8	0.1 (6)	C31—C32—C33—C28	−0.2 (5)
C6—C7—C8—C9	0.6 (6)	C28—P2—C34—C39	86.8 (3)
C7—C8—C9—C4	−0.6 (6)	C22—P2—C34—C39	−164.7 (3)
C5—C4—C9—C8	0.0 (5)	Au1—P2—C34—C39	−32.3 (3)
P1—C4—C9—C8	177.8 (3)	C28—P2—C34—C35	−91.1 (3)
C4—P1—C10—C11	−8.8 (3)	C22—P2—C34—C35	17.5 (3)
C16—P1—C10—C11	−117.7 (3)	Au1—P2—C34—C35	149.9 (3)
Au1—P1—C10—C11	113.0 (3)	C39—C34—C35—C36	−0.6 (5)
C4—P1—C10—C15	178.2 (3)	P2—C34—C35—C36	177.2 (3)
C16—P1—C10—C15	69.4 (3)	C34—C35—C36—C37	−0.9 (6)
Au1—P1—C10—C15	−60.0 (3)	C35—C36—C37—C38	1.0 (6)
C15—C10—C11—C12	0.0 (5)	C36—C37—C38—C39	0.5 (6)
P1—C10—C11—C12	−172.9 (3)	C35—C34—C39—C38	2.1 (5)
C10—C11—C12—C13	−0.5 (5)	P2—C34—C39—C38	−175.8 (3)
C11—C12—C13—C14	0.3 (5)	C37—C38—C39—C34	−2.0 (5)
C12—C13—C14—C15	0.3 (5)		

Symmetry codes: (i) −*x*, −*y*+2, −*z*+2.
